# Can induced headache be used in the differential diagnosis of primary headaches as a diagnostic tool?

**DOI:** 10.1186/1129-2377-14-S1-P141

**Published:** 2013-02-21

**Authors:** Fethi Ýdiman, Gökhan Gürel, FET Ýdiman, GOK Gürel

**Affiliations:** 1 Dokuz Eylül University, Turkey

## 

The objectives of this study are to create headache by visual stimulation(VS) , to examine the similarities between spontaneous headaches and visually stimulated headaches (if the latter occurs), to determine if VS can be used as a model in headache researches and to evaluate if it can be used as a diagnostic tool for the differential diagnosis of primary headaches.

## Methods and materials

In this study, total of 43 migraine patients - 21 with aura and 22 without aura migraine, 21 tension type headache patients and 20 healthy individuals as a control group were included. For VS, a computer simulation was used that was programmed to be 9 HZ frequency color change, 11,2 radius, with the pattern of a yellow-blue dart board and 14 seconds off and 14 seconds on. In order to create headache, stimulation was applied maximum for 45 minutes by using VS.

## Results

The application of VS triggered headache neither in the TTH patients nor in the healthy control subjects. All of the migraine patients were observed to have developed headache. Yet, none was detected to have developed any form of aura. Only two migraine patients without aura did not develop a headache. These two cases were detected to have undergone migraine treatment one day prior to VS. It was observed that the characteristics (location, pattern, and severity) of headache induced by VS in migraine patients and those of spontaneous migraine attack were almost the same. However, headache severity and accompanied signs were also found to be 1-2 points lower in the induced headache with respect to visual analog scale (VAS).

## Conclusion

It was inferred from our study that experimental headache was observed only in migraine patients and shown that as a differential diagnostic tool the method could be applied in the daily practice. It has also been concluded that the VS can be utilized in migraine researches since it is the most almost-natural method.
